# Implications of C1q/TNF-related protein superfamily in patients with coronary artery disease

**DOI:** 10.1038/s41598-020-57877-z

**Published:** 2020-01-21

**Authors:** Yanwei Zhang, Caihong Liu, Jing Liu, Rui Guo, Zheyi Yan, Wenxia Liu, Wayne Bond Lau, Xiangying Jiao, Jimin Cao, Kun Xu, Yongping Jia, Xinliang Ma, Yajing Wang

**Affiliations:** 1 Department of Physiology, Shanxi Medical University, Shanxi, China; 2 Department of Emergency Medicine, Thomas Jefferson University, Philadelphia, PA USA; 3 Department of Cardiology, The First Affiliated Hospital of Shanxi Medical University, Shanxi, China

**Keywords:** Cardiovascular diseases, Endocrinology

## Abstract

The C1q complement/TNF-related protein superfamily (CTRPs) displays differential effects on the regulation of metabolic homeostasis, governing cardiovascular function. However, whether and how they may serve as predictor/pro-diagnosis factors for assessing the risks of coronary artery disease (CAD) remains controversial. Therefore, we performed a clinical study to elaborate on the implication of CTRPs (CTRP1, CTRP5, CTRP7, and CTRP15) in CAD. CTRP1 were significantly increased, whereas CTRP7 and CTRP15 levels were decreased in CAD patients compared to the non-CAD group. Significant differences in CTRP1 levels were discovered between the single- and triple-vascular-vessel lesion groups. ROC analysis revealed that CTRP7 and CTRP15 may serve as CAD markers, while CTRP1 may serve as a marker for the single-vessel lesion of CAD. CTRP1 and CTRP5 can serve as markers for the triple-vessel lesion. CTRP1 may serve as an independent risk predictor for triple-vessel lesion, whereas CTRP15 alteration may serve for a single-vessel lesion of CAD. CTRP1 may serve as a novel superior biomarker for diagnosis of severity of vessel-lesion of CAD patients. CTRP7, CTRP15 may serve as more suitable biomarker for the diagnosis of CAD patients, whereas CTRP5 may serve as an independent predictor for CAD. These findings suggest CTRPs may be the superior predictive factors for the vascular lesion of CAD and represent novel therapeutic targets against CAD.

## Introduction

Coronary artery disease (CAD) is one of the major cardiovascular diseases, which has a serious impact on human health^1^. CAD is a group of diseases including myocardial infarction, sudden angina pectoris, unstable angina pectoris, myocardial infarction, and sudden coronary death^2^. Multiple risk factors for CAD have been identified, which are highly associated with metabolism influencer/regulators including obesity, hypertension, dyslipidemia, diabetes, smoking, gender, age, etc.^3^. However, the sensitivity of the indicators limits the prediction and prognosis of CAD. Identification of new marker or indicator is in great need.

Previous studies suggest that adipokines, well-defined adipocyte endocrine factors, are important determinants for cardiovascular disorders with or without diabetes. Previous investigations implicate adiponectin (APN)’s circulationary level is negatively associated with patients who suffered coronary artery disease and^4^. CTRP (C1q complement/TNF-related protein) as a newly discovered family have shown diversity and wide distribution. Some of CTRP family members have similar metabolic effects with that of adiponectin, whereas others exert opposite effect as that adiponectin. Although emerging evidence suggests that CTRPs may serve as indicators for metabolic disorders, whether and how CTRPs may display its own unique potential relationship in predicting disease, especially cardiovascular disorders, is largely under-investigated.

CTRP family contains 15 family members (CTRP1 to CTRP15). Their functions display the differences and are highly related to their distributions^5^. Of all CTRPs identified to date, CTRP1, CTRP3, CTRP5, CTRP9, CTRP12, CTRP7, and CTRP13 have been reported to exhibit metabolic regulation and cardiovascular effects in animal models^6–8^. CTRP7 circulating levels are reduced in diet-induced diabetic or obese^5,9^, and CTRP7 deletion attenuates obesity-linked glucose intolerance, adipose tissue inflammation, and hepatic stress^10^. CTRP15 is a newly discovered actin that binds skeletal muscle and lipids in response to changes in energy status and predicts changes in metabolic circuits^11^. However, few CTRP members have been investigated in human studies and results are often controversial. For instance, one study reported that CTRP1 may slow the pathogenesis of early atherosclerosis and prevent the development of pathological blood vessels^12^. However, another study demonstrated that CTRP1 level is associated with coronary artery disease^13,14^. CTRP5 level is related to drug-eluting stent implantation after stent restenosis^15,16^. However, the pathogenic or diagnostic role of CTRP5 in coronary diseases remain unclear in CAD patients.

Although above results suggest that CTRPs are associated with metabolism syndromes and play important role in the regulating cellular metabolism,, unclear relationship between circulating CTRPs and coronary artery disease in clinic patients, complexity of CTRP family, and unclear interrelationship between members of CTRP family with different biological function, limited the CTRPs’s clinical application both in therapeutic strategy exploration and as prognostic biomarkers. Therefore, we initiated a clinic investigation on plasma concentrations of CTRP1, CTRP5, CTRP7, and CTRP15, aiming to identify high potential markers to guidance new therapeutic strategy specifically in coronary artery disease, and to predict the severity of CAD. Additionally, the interrelationship of CTRP family in CAD will be assessed.

## Results

### Subjects characteristics

A total of 190 subjects were enrolled in this study. All the demographic data, baseline clinical and biochemical characteristics of the study subjects were summarized in Tables 1 and 2. Statistical analysis showed that CAD patients had markedly higher levels of vascular occlusion, hypertension, diabetes, total cholesterol (TCH), Triglyceride (TG), high density lipoprotein (HDL)-cholesterol, low density lipoprotein (LDL)-cholesterol, alanine aminotransferase (ALT), and aspartate aminotransferase (AST). Interestingly, circulating CTRP1 was significantly high in CAD patients, while CTRP7 and CTRP15 were decreased compared to non-CAD participants (Table 2, p < 0.01). In addition, CTRP7and CTRP15 levels were reduced in elder non-CAD when compared with young non-CAD controls (Table 2).

**Table 1:** Clinical characteristics of participants in CAD and non-CAD groups.

Variable	Non-CAD		CAD			P
	Yong	Elder	Single-vessel-Lesion	Double-vessel-Lesion	Triple-vessel-Lesion	
Sex (M/F)	5/35	17/13	29/11	34/6	28/12	<0.001
Age (year)	34.80 ± 9.73	54.90 ± 8.29	61.13 ± 9.06	63.63 ± 10.61	64.05 ± 8.74	<0.001
Occlusion (N/Y)	40/0	30/0	36/4	36/4	30/10	<0.001
Hypertension (N/Y)	40/0	30/0	24/16	25/15	19/21	<0.001
Diabetes (N/Y)	40/0	30/0	31/9	32/8	28/12	<0.001
Smoker (N/Y)	40/0	27/3	33/7	36/4	34/6	0.106
TCH (mmol/L)	4.49 ± 0.92	4.67 ± 1.15	4.00 ± 1.06	4.07 ± 1.20	4.23 ± 1.21	0.030
Triglyceride (mmol/L)	1.03 ± 0.58	1.66 ± 0.62	1.65 ± 1.12	1.70 ± 0.82	2.17 ± 2.08	<0.001
HDL-cholesterol (mmol/L)	1.43 ± 0.36	1.02 ± 0.15	0.98 ± 0.24	0.92 ± 0.18	1.11 ± 0.76	<0.001
LDL-cholesterol (mmol/L)	2.20 ± 0.62	2.87 ± 0.88	2.29 ± 0.84	2.37 ± 0.96	3.02 ± 2.50	0.009
ALT(U/L)	20.80 ± 23.68	24.10 ± 15.00	28.96 ± 16.11	35.50 ± 37.08	40.98 ± 61.58	<0.001
AST(U/L)	27.87 ± 33.10	21.77 ± 6.48	27.61 ± 13.88	33.58 ± 28.96	38.93 ± 51.47	0.025
EF(%)	63.34 ± 8.72	62.27 ± 9.71	60.58 ± 8.91	60.03 ± 7.45	59.28 ± 9.11	0.197

**p < 0.01 Comparison with non-CAD control subjects. a, p < 0.05 Comparison between single-vessel and triple-vessel.

**Table 2:** Circulating levels of CTRPs of participants in CAD and non-CAD groups.

Variable	Non-CAD		CAD			P
	Yong	Elder	Single-vessel-Lesion	Double-vessel-Lesion	Triple-vessel-Lesion	
CTRP1 (ng/ml)	5.28 ± 3.52	6.69 ± 5.36	6.32 ± 6.22	8.25 ± 9.26	11.31 ± 9.99a**	0.019
CTRP5 (ng/ml)	8.95 ± 3.18	9.82 ± 4.23	14.69 ± 14.36	10.75 ± 4.71	10.26 ± 10.00	0.080
CTRP7 (ng/ml)	44.81 ± 41.02	20.83 ± 23.72**	29.70 ± 51.53**	34.83 ± 53.62**	38.21 ± 78.17**	0.005
CTRP15 (ng/ml)	50.21 ± 34.79	40.40 ± 42.08*	40.40 ± 38.18*	26.82 ± 31.85***	27.29 ± 29.48***	<0.001

**p < 0.01 Comparison with non-CAD control subjects. a, p < 0.05 Comparison between single-vessel and triple-vessel.

To reveal the relationship between CTRPs and vascular lesions and the severity of lesion stages, we evaluated CTRPs levels in different vessel-lesions. Along with increased vascular lesions, CTRP1 and CTRP7 levels increased with the numbers of vessel-lesions in CAD patients (P < 0.05). CTRP5 showed increase tendency in age-matched groups. Notably, CTRP15 levels were decreased in CAD patients, and CTRP15 levels were lower in multiple vessel-lesion group than that in the single-vessel-lesion group. Furthermore, we constructed two models for multivariate regression analysis to assess the independent determinants for severity of diseased coronary vessels. In model 1, we included all risk factors and compared multiple vessel lesions (combining double-vessel and triple-vessel lesion together) to single-vessel lesion. In model 2, we additionally adjusted the analysis by grouping single-vessel and double-vessel as primary lesion and compared with triple-vessel lesion. Both models support that CTRP1 is associated with the severity of vessel-lesion of CAD (Tables 3 and 4). CTRP5 may serve as an independent predictor for CAD.

**Table 3:** Correlation between CTRP1 and number of diseased coronary vessels (multivariate regression analysis).

Variable	OR	OR CI95%	P
CTRP1(ng/ml)	1.049*	1.002–1.097	0.039
Sex(M/F)	1.289	0.461–3.599	0.628
Age(year)	0.998	0.952–1.046	0.934
Hypertension(N/Y)	1.884	0.816–4.347	0.138
HDL-C(mmol/L)	2.060	0.716–5.930	0.180
TCH(mmol/L)	1.152	0.813–1.632	0.426
Smoker(N/Y)	0.755	0.217–2.621	0.658

Comparison single-vessel with double-vessel and triple-vessel lesion.

**Table 4:** Correlation between CTRP1 and number of diseased coronary vessels (multivariate regression analysis).

Variable	OR	OR CI95%	P
CTRP1(ng/ml)	1.066*	1.004–1.132	0.037
Sex(M/F)	0.384	0.132–1.119	0.079
Age(year)	1.041	0.992–1.092	0.106
Hypertension(N/Y)	1.016	0.441–2.343	0.970
HDL-C(mmol/L)	1.180	0.477–2.919	0.720
TCH(mmol/L)	1.151	0.802–1.650	0.446
Smoker(N/Y)	0.362	0.107–1.226	0.102

Comparison single-vessel and double-vessel with triple-vessel lesion.

To further clarify the relationship between CTRPs and CAD, we performed a serial analysis. First, as illustrated in Table 5, we build a multivariate model among the significant variables noted by univariate analysis to determine potential screening markers for CAD. Results showed that CAD is highly correlated with Sex, age, diabetes and AST (p < 0.05). CTRP5 was an independent predictor for CAD (Table 5). Meanwhile, Sex, age, diabetes, smoking, TCH, TG, HDL-C, ALT, CTRP1 and CTRP15 were significantly positively associated with CAD (Table 6). Logistic regression analysis as shown in Table 7 and 8 indicated that reduced CTRP15 was a high-risk factor for a single-vessel lesion in CAD whereas increased CTRP1, occlusion and smoking were the high-risk factors for triple-vessel lesions of CAD.

**Table 5:** Correlation between CAD and CTRPs (multivariate regression analysis).

Variable	OR	OR CI95%	P
Sex	8.92**	1.91–41.76	0.005
Age	0.82***	0.74–0.89	<0.001
Diabetes	207.82**	5.53–7811.55	0.004
CTRP5	1.17*	0.73–0.99	0.038
AST	0.96*	0.93–0.99	0.029

*p < 0.05. **p < 0.01. **p < 0.001.

**Table 6:** Logistic regression analysis of related risk factors. for CAD.

Variable	Univariate Analysis		
	OR	ORCI95%	P
Sex(M/F)	6.846***	3.56–13.18	<0.001
Age(year)	1.15**	1.11–1.20	0.001
Diabetes(N/Y)	21.99**	2.92–165.41	0.003
Smoker(N/Y)	3.69*	1.04–13.07	0.043
TCH(mmol/L)	0.69**	0.53–0.90	0.007
TG(mmol/L)	1.97**	1.27–3.06	0.002
HDL-C(mmol/L)	0.17**	0.06–0.47	0.001
LDL-C(mmol/L)	1.04	0.83–1.31	0.710
ALT(U/L)	1.03*	1.01–1.05	0.011
AST(U/L)	1.02	0.10–1.03	0.114
EF(%)	0.97	0.92–1.02	0.198
CTRP1(ng/ml)	1.06*	1.01–1.12	0.022
CTRP5(ng/ml)	1.05	0.10–1.11	0.058
CTRP7(ng/ml)	1.00	0.99–1.01	0.967
CTRP15(ng/ml)	0.99**	0.98–0.10	0.009

**p < 0.01.*p < 0.05. **p < 0.001.

**Table 7:** Logistic regression analysis of related risk factors for single-vessel of CAD.

Variable	Univariate Analysis		
	OR	ORCI95%	P
Sex(M/F)	1.307	0.547–3.119	0.547
Age(year)	0.970	0.931–1.010	0.143
Occlusion(N/Y)	0.524	0.160–1.710	0.284
Hypertension(N/Y)	0.815	0.377–1.761	0.603
Diabetes(N/Y)	0.871	0.355–2.138	0.763
Smoker(N/Y)	1.485	0.519–4.247	0.461
TC(mmol/L)	0.890	0.636–1.246	0.498
TG(mmol/L)	0.834	0.586–1.186	0.312
HDL-C(mmol/L)	0.822	0.331–2.044	0.674
LDL-C(mmol/L)	0.792	0.548–1.146	0.216
ALT(U/L)	0.992	0.976–1.007	0.287
AST(U/L)	0.987	0.967–1.008	0.219
EF(%)	1.013	0.968–1.061	0.572
CTRP1(ng/ml)	0.945	0.892–1.000	0.051
CTRP5(ng/ml)	1.039	0.997–1.083	0.067
CTRP7(ng/ml)	0.998	0.991–1.005	0.571
CTRP15(ng/ml)	1.011*	1.000–1.023	0.045

*p < 0.05.

**Table 8:** Logistic regression analysis of related risk factors for triple-vessel of CAD.

Variable	Univariate Analysis		
	OR	ORCI95%	P
Sex(M/F)	1.588	0.670–3.763	0.293
Age(year)	1.019	0.978–1.061	0.363
Occlusion(N/Y)	3.000*	1.079–8.341	0.035
Hypertension(N/Y)	1.747	0.812–3.760	0.154
Diabetes(N/Y)	1.588	0.670–3.763	0.293
Smoker(N/Y)	3.69*	1.04–13.07	0.043
TC(mmol/L)	1.162	0.837–1.613	0.370
TG(mmol/L)	1.278	0.945–1.727	0.112
HDL-C(mmol/L)	2.170	0.768–6.131	0.144
LDL-C(mmol/L)	1.346	0.974–1.859	0.071
ALT(U/L)	1.005	0.996–1.014	0.391
AST(U/L)	1.007	0.995–1.018	0.263
EF(%)	0.986	0.92–1.02	0.531
CTRP1(ng/ml)	1.052*	1.006–1.099	0.025
CTRP5(ng/ml)	0.971	0.925–1.020	0.243
CTRP7(ng/ml)	1.002	0.996–1.007	0.621
CTRP15(ng/ml)	0.994	0.982–1.006	0.334

*p < 0.05.

**Figure 1:**
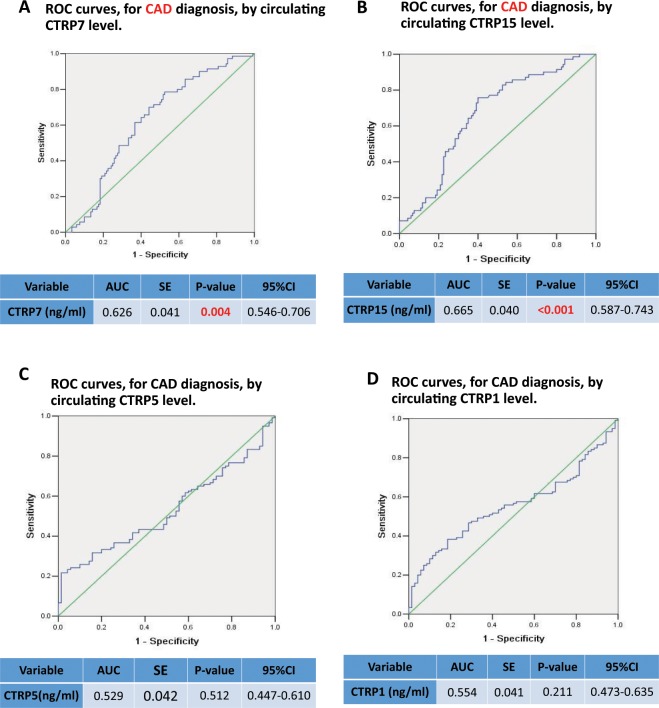
ROC curves for CAD diagnosis by CTRPs level. (**A**) ROC curves for CAD diagnosis by CTRP7 level. (**B**) ROC curve for CAD diagnosis by circulating CTRP15 level. (**C**) ROC curve for CAD diagnosis by CTRP5 level. (**D**) ROC curve for CAD diagnosis by CTRP1 level.

**Figure 2:**
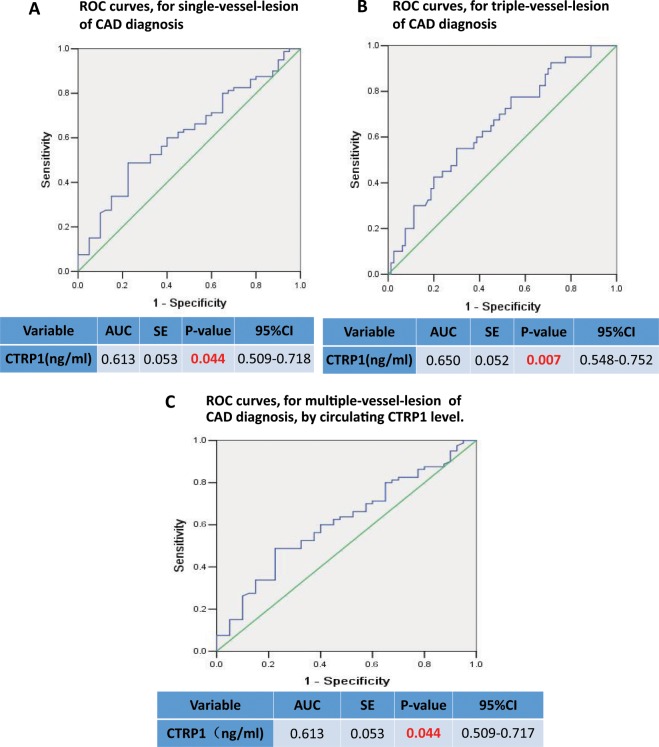
ROC curves for vessel-lesion of CAD diagnosis by CTRP1 level. (**A**) ROC curve for single-vessel-lesion of CAD diagnosis by CTRP1 level. (**B**) ROC curve for triple-vessel-lesion of CAD diagnosis by CTRP1 level. (**C**) ROC curve for multiple-vessel-lesion of CAD diagnosis by CTRP1 level.

**Figure 3:**
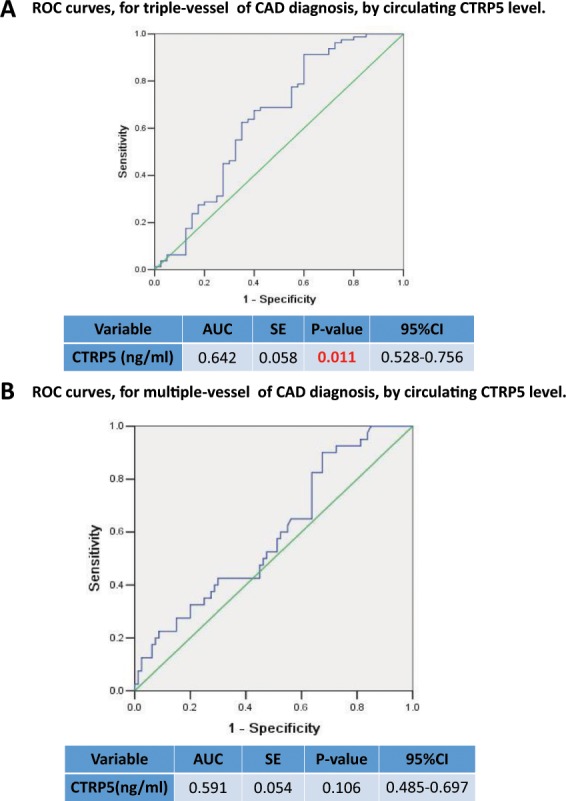
ROC curves for vessel-lesion of CAD diagnosis by CTRP5 level. (**A**) ROC curve for triple-vessel-lesion of CAD diagnosis by CTRP5 level. (**B**) ROC curve for multiple-vessel-lesion of CAD diagnosis by CTRP5 level.

To assess whether CTRPs can serve as a marker for CAD, a receiver operating characteristic (ROC) curve was constructed to evaluate the diagnostic value of CTRPs for CAD. As illustrated in Fig. 1, the AUC (area under the curve) was 0.626 for CTRP7 (P < 0.01, Fig. 1A, n = 120) and 0.665 for CTRP15 (p < 0.001, Fig. 1B, n = 120), confirming specificity and sensitivity of CTRP7 and CTRP15 as biomarkers for CAD. The ROC analysis results of CTRP5 and CTRP1 showed the AUC was 0.529 and 0.554 respectively, failed to serve as biomarkers for CAD as a whole. Notably, the AUC of CTRP1 was 0.613 (95% confidence interval 0.509–0.718, P = 0.044) for single-vessel lesions (Fig. 2A), and AUC was 0.650 (p = 0.007) for triple-vessel (Fig. 2B). Furthermore, the AUC of CTRP1 for multiple-vessel lesions was 0.613 (p = 0.044) (Fig. 2C), hence, CTRP1 value was the optimistic selected models for prognosticating vessel-lesions in CAD. Although the AUC of CTRP5 for triple-vessel lesions was 0.642 (p = 0.011), the signal-vessel lesion and multiple-vessel lesion failed to serve as a marker (Fig. 3). Taken together, these results suggest that CTRP7 and CTRP15 may serve as biomarkers for CAD as whole while CTRP1 may serve as a marker for severity of vessel-lesions.

To identify the etiology of altered CTRPs in CAD patients, we examined whether CTRPs is associated with other risk factors. Multiple linear regression was used to determine the relationship of CTRPs with other risk factors. As shown in Tables 9 and 10, the results revealed that CTRP5 and CTRP7 were not associated with any other variables. However, serum CTRP1 was positively associated with age and vascular occlusion. When other variables remain the same, CTRP1 increases by 0.088 ng/ml for each additional year of age (Table 9). Meanwhile, there was no evidence revealing the positive interrelationship among the CTRP family members in CAD population.

**Table 9:** Multiple linear regression analysis of CTRP1 and clinical indicators.

Variable	β	SE	P
Sex(M/F)	0.984	1.248	0.431
Age(year)	0.088*	0.042	0.037
Occlusion(N/Y)	4.445*	1.889	0.020
Diabetes(N/Y)	0.733	1.698	0.666
TCH(mmol/L)	−0.531	0.546	0.332
HDL-C(mmol/L)	1.253	1.326	0.346
Smoker(N/Y	2.791	1.903	0.144
AST(U/L)	0.022	0.017	0.202
TG(mmol/L)	0.906	0.506	0.075
Hypertension(N/Y)	0.033	1.526	0.983

*p < 0.05.

**Table 10:** Multiple linear regression analysis of CTRP15 and clinical indicators.

Variable	β	SE	P
Sex(M/F)	4.222	5.832	0.470
Age(year)	−0.606**	0.196	0.002
Occlusion(N/Y)	2.675	8.828	0.762
Diabetes(N/Y)	−19.379*	7.935	0.016
TCH(mmol/L)	6.162*	2.553	0.017
HDL-C(mmol/L)	−5.271	6.197	0.396
Smoker(N/Y)	4.577	8.893	0.607
AST(U/L)	−0.132	0.081	0.104
TG(mmol/L)	−0.990	2.365	0.676
Hypertension(N/Y)	11.924	7.134	0.096

*p < 0.05.

In contrast, age, diabetes, and TCH had a significant association with CTRP15. Serum CTRP15 was inversely correlated with age and diabetes, but TG was positively correlated with CTRP15. In order to further determine the relationship between CTRPs and severity of vessel-lesions in coronary artery disease, we have studied the correlation between the number of lesions in CAD and clinical indicators. The results showed that CTRP1 level is positively correlated with AST in single-vessel disease (Table 11). CTRP15 and diabetes were negatively correlated with the three-vessel disease (Table 12).

**Table 11:** Multiple linear regression analysis of CTRP1 and clinical indicators in single-vessel.

Variable	β	SE	P
Sex(M/F)	−0.705	2.685	0.795
Age(year)	0.119	0.138	0.395
Occlusion(N/Y)	0.406	3.626	0.912
Diabetes(N/Y)	2.589	2.413	0.292
TCH(mmol/L)	−0.049	1.276	0.969
HDL-C(mmol/L)	1.745	5.786	0.765
Smoker(N/Y	4.298	2.935	0.154
AST(U/L)	0.168*	0.079	0.044
TG(mmol/L)	0.777	1.056	0.468
Hypertension(N/Y)	1.593	2.256	0.486

*p < 0.05.

**Table 12:** Multiple linear regression analysis of CTRP15 and clinical indicators in triple-vessel.

Variable	β	SE	P
Sex(M/F)	12.408	13.942	0.381
Age(year)	−0.723	0.829	0.390
Occlusion(N/Y)	−3.458	13.430	0.799
Diabetes(N/Y)	−32.302*	15.032	0.040
TCH(mmol/L)	0.190	5.196	0.971
HDL-C(mmol/L)	−2.816	7.637	0.715
Smoker(N/Y)	2.251	17.093	0.896
AST(U/L)	−0.092	0.128	0.478
TG(mmol/L)	0.145	3.202	0.964
Hypertension(N/Y)	25.093	14.429	0.093

## Discussion

We reported a discovery of new biomarkers for coronary artery disease. The current study demonstrated that CTRP7 and CTRP15 may serve novel biomarkers of CAD, and CTRP1 may predict the vessel-lesion severity in CAD. Moreover, CTRP5 may serve an independent predictor for CAD. In addition, compared with the control group, concentrations of both CTRP5 and CTRP7 were significantly decreased in CAD patients while CTRP1 concentrations were elevated.

In recent years, there have been many studies investigating serum levels of CTRP1 and coronary artery disease. Studies have shown that CTRP1 is a marker of human atherosclerosis and promotes atherosclerosis in mice^17–19^. The serum of the three-vessel disease had a high concentration of CTRP1 than the single-vessel disease, which was consistent with our findings. Our results indicated that compared with non-CAD people, the serum level of CTRP1 was elevated and correlated with CAD. CTRP1 highly correlated with the stages of vessel-lesion. Although CTRP1 was a significant factor for predicting vessel-lesion stages in CAD, it failed to be a diagnostic biomarker to CAD. This is inconsistence with previous report by other group^13^. Small patients’ populations and limited race (Chinese Han population) could be the reason to cause this discrepancy. However, it acts as a biomarker for the increase of the vessel-lesion level. The increased level of CTRP1 in circulation may be attributed to disturbed flow and other risk factors-induced inflammatory cytokines^20,21^. To further explore the risk factor for elevated CTRP1 and elaborate the relationship between clinical indicators and CTRP1, we applied multiple linear regression assay. The results indicated that plasma CTRP1 levels were positively correlated with age and vascular occlusion. Furthermore, CTRP1 was positively correlated with AST in single-vessel disease. Despite the advantageous findings of our study regarding the role of CTRP1 in CAD related vessel lesion, we recognize some limitations. We did not investigate other vascular disease relationship with CTRP1 and other possible pathogenic molecules including other CTRP family members. It is highly desirable to conduct additional studies regarding CTRP1 role in pathogenesis of CAD with larger samples of patients utilizing prospective designs.

CTRP5 is wildly expressed in various cells, with the highest expression level in adipose tissue^22^. It inhibits adiponectin and resistin release in a dose-dependent manner^16^. Simultaneously, CTRP5 can promote transcytosis and oxidative modification of low-density lipoprotein and accelerate atherosclerosis development^23^. Studies have shown that CTRP5 can be used as a biomarker for potential new inflammatory chronic obstructive pulmonary disease and in patients with coronary in-stent restenosis since serum CTRP5 levels are significantly elevated^24^. However, there are few studies investigated the relationship between CTRP5 and coronary artery disease. In our study, we divided the recruited patient’s population into two groups (elder and young) in the normal physical examination group. Three stages of vessel-lesion in diagnosed as coronary artery disease group and the CTRP5 levels were compared among the groups. The results showed no differences within the non-CAD group in different age. However, CTRP5level was increased in CAD group when compared with non-CAD. Notably, the concentration of serum CTRP5 shown an increasing tendency in a single-vessel lesion group than the three-vessel lesion group. The small sample size and the controversial deleterious effect of CTRP5 in the vascular system may contribute to the unparalleled phenomenon between CTRP5 and the level of vessel lesions^16,23,25^.

Although circulating CTRP7 level has been reported to be elevated in obese people, and CTRP7 knockout mice exhibit elevated glucose metabolism, decreased adipose tissue inflammation, liver fibrosis, cellular oxidation, and endoplasmic reticulum stress in^10^, the CTRP7 circulating levels and coronary artery disease is largely unknown. Studies have shown that circulating CTRP15 concentration and its gene expression are affected by metabolism^26^. CTRP15 may potentially be used as a circulating biomarker for predicting cardiovascular disease (CVD) risk in obese and T2DM patients^27^. In our study, serum CTPR7 and CTRP15 levels in CAD were reduced compared with the non-CAD group. CTRP7 and CTRP15 are novel identified diagnostic biomarker for CAD, while CTRP1 and CTRP5 fail in this study. These results suggest that CTRP7 and CTRP15 presents more sensitivity and stability as biomarkers than the other two. Meanwhile, the result showed that CTRP15 was negatively correlated with age and diabetes in coronary artery disease patients; and it is positively correlated with TG levels. Further analysis revealed that the negative correlation between CTRP15 and diabetes is significant in the three-vessel lesion of CAD.

We further carried out multiple regression analysis and found that CTRP5 can be an independent indicator for CAD, but it failed to predict the severity of vessel-lesion, and CTRP5 might be a possible therapeutic target in prevention or ameliorating CAD in future study. In addition, consistent with other studies^28^, gender, age, diabetes, and AST are all risk factors for CAD.

Our research has some limitations. The first is relatively small sample size and is Chinese Han population only. Secondly, our survey was a case-controlled study by which it cannot determine the incident rate of disease in exposed and non-exposed groups. Thirdly, we utilized AUC >0.6 as cutoff for the resultant logistic-regression based ROC analyses. However, CTRPs are not pre-existing risk model in CAD and our study was restricted to a small study size. This factors AUC has value for evaluating CAD risks even with a cutoff value of 0.6. Fourthly, mechanisms causing circulating CTRPs level alteration need to be further explored. Our preliminary experiments suggest that unbalanced miRNA pool produced by dysfunctional adipocytes may initiate pathological long-distance communication between adipocytes and cardiomyocytes, causing CTRPs level alteration, abnormal CTRP9 assembling and expression. Finally yet importantly, we needed additional *in vivo* animal studies to elucidate the physiology and mechanism of action of CTRP in the development of CAD.

In conclusion, we have demonstrated that circulating CTRP1 can predict the severity of vessel-lesions in coronary artery disease, and CTRP7 and CTRP15 can serve as new CAD diagnostic biomarker. In addition, our study showed that CTRP15 is inversely associated with diabetes in three-vessel coronary artery disease. CTRP5 is the independent risk for CAD. The new discovered CTRPs provide the future therapeutic target and pre-diagnostic biomarker for coronary artery disease.

## Methods

### Participants and eligibility

The patients were enrolled in the first affiliated Hospital of Shanxi Medical University from January 2017 to September 2018. A total of 190 subjects attended. The research included 40 healthy control subjects, 30 patients without stenosis after coronary angiography, and 120 patients with coronary artery disease (40 patients with a single vessel, 40 patients with double-vessel and 40 patients with triple-vessel). All research protocols were approved by the Ethics Committee of Shanxi Medical University and performed in accordance with the latest version of the Declaration of Helsinki. Moreover, all patients and control subjects were given written informed consent. The diagnostic criteria of CAD were a 50% or greater organic stenosis of at least one main coronary artery as confirmed by coronary angiogram, two stenosis ≥50% was considered as a double-vessel disease, and more than two stenoses was defined as a triple-vessel disease. Exclusion criteria for this study were: old myocardial infarction, congestive heart failure, severe hepatic and renal dysfunction, malignancy, a history of trauma or surgery within one-month, valvular heart disease, pregnancy, or any factor affecting body weight such as hyperthyroidism, corticosteroids, or contraceptives, or acute and chronic infection.

### Biochemical and hormonal analysis

The arterial blood extracted directly from the sheath of radial artery before coronary angiography was separated into the Eppendorf tube at 4 °C for 20 minutes by 3000 rpm centrifugation, then frozen and stored at −80 °C until determined. Total cholesterol(TC), triglycerides(TG), high-density lipoprotein (HDL) cholesterol, low-density lipoprotein (LDL) cholesterol, homocysteine (HCY) were determined by commercial kit via Hitachi 7600 biochemical automatic analyzer (Hitachi, Tokyo, Japan).

### Echocardiographic examination

The echocardiographic measurements were carried out while the patients were in the left lateral decubitus with standard precordial positions by Philips Epiq 7C Cardiology Ultrasound machine (Los Angeles, CA, USA). The left ventricle ejection fraction (LV EF) was assessed with biplane method of disks (modified Simpson’s rule) according to the American Society of Echocardiography (ASE). LVEF = [(LV end-diastolic volume)- (LV end-systolic volume)]/ (LV end-diastolic volume).

### Measurement of plasma CTRP1, CTRP5, CTRP7 and CTRP15

Plasma CTRP1, CTRP5, CTRP7, and CTRP15 levels were measured by commercial enzyme-linked immunosorbent assay (ELISA) kit (Cat, SK00083-01 for CTRP1; Cat, SK00594-06 for CTRP5; Cat, SK00396-09 for CTRP7; SK00393-19 for CTRP15, Aviscera Bioscience, Santa Clara, CA) per manufacturer’s instructions. Intra- and inter-assay coefficients of variation (CV) for CTRP1, CTRP5, and CTRP15 were 6–8% and 8–12% respectively, and intra- and inter-assay coefficients of variation for CTRP7 were 4–6% and 8–10%, respectively.

### Statistical analysis

Descriptive analysis was applied, and quantitative data were conducted by the Shapiro-Wilk test for normality. Continuous variables are expressed as mean ± standard error (SE). Data (Sex, Smoker, Diabetes) that did not conform to normality distribution were expressed as median ± interquartile (IQR). We first examined differences between groups of the phenotypes of interest in the control, single-vessel-lesion, double-vessel-lesion, and triple-vessel-lesion by the Mann-Whitney U test to compare of continuous variables and the Chi-square test for statistical inference of categorical variables. To assess the effect of the subjects with different lesions, we applied a Chi-square test on different groups.

Next, we used logistic regression to estimate the association between CAD and case-control status of all other parameters as defined above. In addition to the use of multivariate regression for determining potential screening markers for CAD, we adjusted our model for other risk factors by univariate regression analysis and evaluated the collinearity diagnostics. The predicted probability of being diagnosed with CAD was used as a surrogate marker to construct receiver operating characteristic (ROC curves). The area under the ROC curve (AUC) served as an accuracy index evaluating the diagnostic performance of the noted marker. We used multiple linear regression to describe the dependencies between CTRPs and multiple independent variables in CAD.

All statistical analyses were performed using SPSS 20.0 (Chicago, IL, USA). P-value less than 0.05 in a two-tailed test were considered statistically significant.
